# Mixed Acinar Neuroendocrine Carcinoma of the Pancreas: Comparative Population-Based Epidemiology of a Rare and Fatal Malignancy in The United States

**DOI:** 10.3390/cancers15030840

**Published:** 2023-01-30

**Authors:** Amro M. Abdelrahman, Jun Yin, Roberto Alva-Ruiz, Jennifer A. Yonkus, Jennifer L. Leiting, Isaac T. Lynch, Alessandro Fogliati, Nellie A. Campbell, Danielle M. Carlson, Lewis R. Roberts, Gregory J. Gores, Rory L. Smoot, Rondell P. Graham, Thorvardur R. Halfdanarson, Mark J. Truty

**Affiliations:** 1 Division of Hepatobiliary and Pancreas Surgery, Mayo Clinic, Rochester, MN 55905, USA; 2 Division of Clinical Trials and Biostatistics, Mayo Clinic, Rochester, MN 55905, USA; 3 Division of Gastroenterology and Hepatology, Mayo Clinic, Rochester, MN 55905, USA; 4 Division of Anatomic Pathology, Mayo Clinic, Rochester, MN 55905, USA; 5 Division of Medical Oncology, Mayo Clinic, Rochester, MN 55905, USA

**Keywords:** MANEC-P, epidemiology, rare cancers, mixed carcinoma, cancer surveillance and screening

## Abstract

**Simple Summary:**

Mixed acinar neuroendocrine carcinoma of the pancreas (MANEC-P) is one of the rarest pancreatic carcinomas. Although MANEC-P is rare, it is associated with a bad prognosis. The distribution of this fatal cancer is unknown worldwide. Our contribution is expected to be a detailed understanding of the epidemiology of this cancer in the United States. Expected results from the proposed research include an estimation of the incidence, prevalence, and cancer-specific survival of patients diagnosed with MANEC-P from a population-based cancer registry. This study will be followed by multiple epidemiological studies on the distribution of this fatal cancer worldwide. These epidemiological benchmarks will be used to initiate a consortium to generate real-world evidence with a natural history study for risk identification and treatment optimization for MANEC-P. The long-term goal is to develop an up-to-date epidemiologic and multi-omics knowledge library to enhance the diagnostics and therapeutics options and improve survival among patients diagnosed with MANEC-P.

**Abstract:**

Mixed acinar neuroendocrine carcinoma of the pancreas (MANEC-P) is an extremely rare malignancy with a poor prognosis. However, epidemiological estimates of MANEC-P remain unknown. This study aimed to estimate and compare the incidence, prevalence, and cancer-specific survival (CSS) of MANEC-P in the United States (US). Patients with MANEC-P were identified through the Surveillance, Epidemiology, and End Results (SEER) and National Program of Cancer Registries databases between 2000–2017. The primary outcomes included age-adjusted incidence rate, limited-duration prevalence, and CSS. A total of 630 patients were identified for the incidence analysis and 149 for the prevalence and CSS analyses. The MANEC-P incidence rate was 0.011 per 100,000 individuals, which was the lowest among pancreatic cancer histologic subtypes. The incidence rate was significantly higher in men and Black races and peaked at 75–79 years of age. The incidence rate was the lowest in the midwestern region (0.009) and the highest in the northeastern US (0.013). The 17-year prevalence was 0.00005%, indicating that 189 patients were alive in the United States at the beginning of 2018. The median CSS of MANEC-P was estimated to be 41 (23, 69) months. In conclusion, MANEC-P is very rare, and its incidence rate has been steady in the US over the last two decades. MANEC-P has a poor prognosis and is the 5th leading cause of pancreatic cancer-related death in the US.

## 1. Introduction

Mixed acinar neuroendocrine carcinoma of the pancreas (MANEC-P) is a unique cancer composed of acinar exocrine and endocrine components, comprising at least 25–30% of each cellular component [1,2]. MANEC-P is among the rarest histologic subtype of pancreatic cancer [3]. It is difficult to diagnose [4,5,6,7] and has a poor prognosis [8,9,10]. A study utilizing the National Cancer Database (NCDB) reported that the 5-year overall survival of MANEC-P was 37% [11], with a median overall survival ranging between 17 and 27 months [11,12], making MANEC-P one of the leading causes of pancreatic cancer-related deaths [11,13]. However, overall survival data are largely collated from sporadic case reports and small case series [12,14,15,16,17,18]. Although other studies have conveyed prognostic data of rare pancreatic subtypes using the National Cancer Database (NCDB) [11], the NCDB database only reports overall survival. Fundamental epidemiological measures, including the incidence, prevalence, and cancer-specific survival (CSS) of MANEC-P are not available. There remains a gap in epidemiological knowledge regarding this fatal cancer, as these measures have not been estimated or compared to other pancreatic cancer histological subtypes. Without such detailed epidemiological estimates of MANEC-P, the ability to decrease MANEC-P-related mortality is likely to remain limited.

Comprehensive epidemiological studies on MANEC-P are needed to establish benchmarks for future diagnostic and therapeutic studies. This study aimed to identify epidemiological measures of MANEC-P using the Surveillance, Epidemiology, and End Results (SEER) Program Cancer Registry and the Center for Disease Control and Prevention’s (CDC) National Program of Cancer Registries (NPCR) and provide a detailed understanding of the descriptive and comparative epidemiology of MANEC-P in the United States (US). This is a significant contribution because it is expected to provide the foundation for future comprehensive natural history studies accompanied by translational and clinical studies to improve MANEC-P long-term outcomes in patients afflicted with this rare cancer type.

This study aimed to estimate the incidence, prevalence, and CSS of MANEC-P and compare them with other pancreatic cancer histological subtypes in the US. With such an in-depth epidemiological investigation, resources will be effectively directed toward well-planned studies to evaluate MANEC-P clinical and biological evolution, patient quality of life, and detailed therapeutic modalities to identify effective and safe therapeutics to improve MANEC-P oncological outcomes.

## 2. Materials and Methods

Research Setting and Design: This was a population-based retrospective analysis of NPCR and SEER databases. 

Study Population: This study included all patients diagnosed with MANEC-P in the United States. For incidence, patients were included between 2001 and 2017 through the following dataset: “NPCR and SEER Incidence—U.S. Cancer Statistics 2001–2017 Public Use Research Database, 2019 Submission (2001–2017), US Department of Health and Human Services, Centers for Disease Control and Prevention (CDC), and the National Cancer Institute (NCI), released in June 2020 and accessed on 12 August 2020 at www.cdc.gov/cancer/uscs/public-use”.

For prevalence and CSS, SEER had four primary datasets developed in chronological order depending on the number of states participating in SEER over the years: SEER9, SEER13, SEER18, and SEER21 (the number represents the number of states reporting to that dataset). The SEER9 dataset is the oldest and has captured cancer cases since its inception in 1975. SEER 21 is the newest and has more participating states but has only captured cases since 2000. We extracted subjects from all four datasets but identified only three reported MANEC-P cases before 2000. Therefore, we excluded SEER9 and SEER13, and narrowed our study focus between the years 2000–2017. We excluded SEER21 because it did not contain all relevant study variables. SEER18 was included in the data analysis, specifically, the SEER database, “Incidence—SEER 18 Regs Custom Data (with additional treatment fields), Nov 2018 Sub (1975–2016 varying)—Linked To County Attributes—Total U.S., 1969–2017 Counties, National Cancer Institute, DCCPS, Surveillance Research Program, released April 2019, based on the November 2018 submission”. 

Data Collection Methods: The NPCR provides data from 46 states, the District of Columbia, Puerto Rico, the U.S. Pacific Island Jurisdictions, and the U.S. Virgin Islands, which is approximately 97% of the U.S. population [19]. The SEER database includes more than 28 population-based tumor registries, encompassing approximately 48% of the US population [20]. Combined NPCR and SEER data provide cancer incidence and population data for the District of Columbia, Puerto Rico, and all 50 states, with information on more than 26 million cancer cases [21]. MANEC-P cases were identified using the ICD-O-3 topographic range C25.0–C25.9, and ICD-O-3 morphologic and behavior code M-8154/3. Only patients with positive histology were included in the study. Patients with MANEC-P with a primary site other than the pancreas were excluded from the study. Table S1 shows the ICD-O-3 morphology and behavior codes used to identify other histological subtypes of pancreatic cancer for comparative epidemiological analysis. For comprehensive ICD-O-3 codes, we used detailed SEER resources and ICD-O-3 manuals [22].

Dependent and independent variables: Patient characteristics evaluated and collected included demographic data (i.e., age at diagnosis, sex, ethnicity, race, health service area, state, and year and month of diagnosis) and tumor characteristics (i.e., site, size, grade [I, II, III, and IV], historical staging, and multiple primaries). Age at diagnosis was categorized into 5-year categories except for the 00–39 years-old group and the 85+-year-old groups. Tumor sites included the head, body, tail, islets of Langerhans (IOL), other specified parts, overlapped lesions, and not otherwise specified (NOS). Historical tumor staging was categorized into the following groups: in situ, localized disease, regional by direct extension only, regional lymph nodes involved only, regional by both direct extension and regional lymph nodes involved, regional NOS, distant site(s)/node(s) involved, not applicable, or unknown/unstaged/unspecific. Multiple primary site variable describes the order of MANEC-P in case of other multiple tumors, including the following categories: one primary only, first of two or more primaries, second of two or more primaries, and third of three or more primaries.

Primary outcomes included: (1) incidence, (2) prevalence, and (3) cancer-specific survival (CSS). The incidence was defined as newly diagnosed cases of MANEC-P occurring annually. The annual population denominators were obtained from the SEER-Stat. Prevalence was defined as the number of MANEC-P patients who were alive on a specific date among a population that previously had MANEC-P. Cancer-specific survival was defined as MANEC-P survival in the absence of other causes of death. This was calculated using a standard life table approach with the SEER cause-specific survival death classification, in which individuals who died of reasons other than MANEC-P were censored. Cumulative MANEC-P-specific survival rates were also calculated. 

Data Analysis Methods: Descriptive analyses were conducted to report patient demographics, tumor characteristics, and treatment modality differences among patients with MANEC-P. Incidence was estimated as newly diagnosed cases per 100,000 population during a specific period. Differences between groups were reported using Student’s *t*-test, Fisher’s exact test, covariate (ANOVA), and Kruskal–Wallis test, as appropriate. The annual population denominators were obtained from SEER-Stat. Annual percentage change (APC) and trend analyses were conducted using joinpoint regression analysis [23]. Limited-duration prevalence was calculated using estimates of persons living with MANEC-P at the time of the analysis since 2000. CSS was estimated using the Kaplan–Meier method. These epidemiological measures were age-adjusted to the 2000 U.S. standard population. The incidence and CSS of all pancreatic cancer subtypes were identified and compared to those of MANEC-P. Statistical significance was set at *p* < 0.05.

All analyses were performed using the SEER-Stat, Joinpoint 4.8.0.1 (Joinpoint Regression Program, Version 4.8.0.1. April 2020 Statistical Research and Applications Branch, National Cancer Institute, Bethesda, MD, USA), HD*Calc Version 2.0.0 (Surveillance Research Program, National Cancer Institute, Bethesda, MD, Health Disparities Calculator Software (www.surveillance.cancer.gov/disparities, accessed on 12 August 2020) version 2.0.0), ProjPrev Version 1.0.6 (Information Management Services, Inc. in consultation with The data Modeling Branch of the Cancer Control and Population Sciences Division of the National Cancer Institute (NCI), Bethesda, MD, https://surveillance.cancer.gov/projprev/, accessed on 12 August 2020), JMP Pro 14.1.0 (SAS Institute Inc., Cary, NC, USA) software packages.

## 3. Results

### 3.1. Incidence Rate

**Figure 1:**
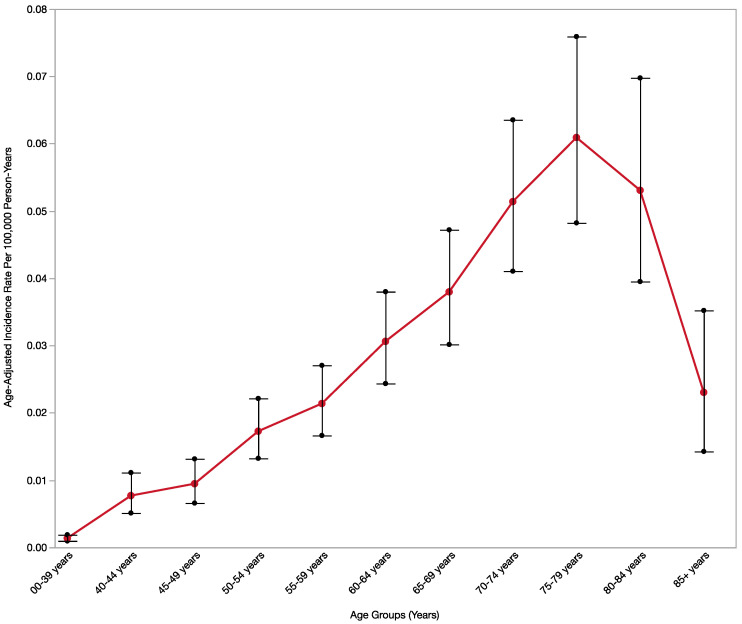
MANEC-P incidence rate by age groups. The red dots and line connect the age-adjusted incidence rate per 100,000 person-years for each age group and the black bars are the 95% confidence interval around each age-adjusted incidence rate for each age group.

Demographic Trend: A total of 630 patients were identified for incidence analysis through NPCR and 149 for prevalence and CSS analysis through SEER. Table 1 shows the MANEC-P incidence rate distribution based on demographics, tumor characteristics, and temporal trends. The incidence rate of MANEC-P was 0.011 per 100,000 population. The incidence rate increased with increasing age and peaked (0.061) in the 75–79 to years-old patient group (Figure 1). The incidence was higher in men than in women (0.013 vs. 0.009, *p* < 0.05). The incidence rate by sex remained significant in the two age groups: 65–69 and 75–79 years-old patients (Figure S1). The incidence of MANEC-P was significantly higher in the non-Spanish-Hispanic-Latin group (0.012 vs. 0.008, *p* < 0.05). Although most MANEC-P patients were white, the incidence rate was slightly higher in the Black population (0.013) and lower in the Asian or Pacific Islander population (0.009) than in the white population (0.011). However, these differences were not statistically significant (Table 1). 

At diagnosis, the MANEC-P incidence rate was highest in the head of the pancreas (0.004), followed by the tail and body of the pancreas, with significantly lower incidence rates (0.003 and 0.001, respectively; both *p* < 0.05). Although most MANEC-P cases were not graded, MANEC-P incidence rates were similar (approximately 0.002) for all grades. However, the incidence rate of grade IV was significantly lower than that of grade I (0.0003, *p* < 0.05). There were no sex differences between MANEC-P grade I and II incidence rates; however, grade III MANEC-P patients were predominantly male (Figure S2). 

When using the historical staging system to compare in situ versus localized MANEC-P disease, there was a higher incidence of the distant site/nodal disease (0.004 vs. 0.002, *p* < 0.0001), and a significantly lower incidence rate of regional lymph node disease only (0.0009 vs. 0.002, *p* < 0.0001).

The incidence rate was higher when MANEC-P was reported as the sole primary tumor than when it was reported as the second of two reported tumors (0.008 vs. 0.002; *p* < 0.05). Compared to the incidence rate of MANEC-P as the only primary tumor, MANEC-P was rarely reported as the first of two tumors or the third of three tumors (0.0008 and 0.0002, respectively; *p* < 0.05).

**Figure 2:**
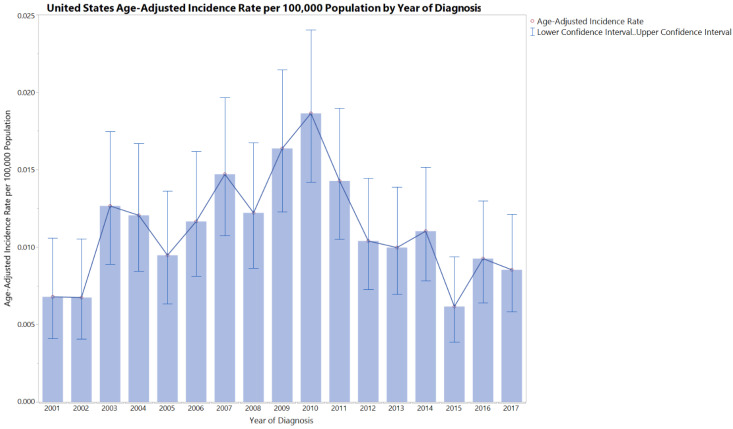
United States Temporal Trend over the time between 2001 and 2017 shows the age-adjusted incidence rate per 100,000 for each year of patients diagnosed with mixed pancreatic endocrine and exocrine tumor, malignant. The error bars are between the lower and the upper 95% confidence interval of the age-adjusted incidence rate.

Descriptive Temporal and Seasonal Trend: Figure 2 shows the MANEC-P incidence rate distribution by year of diagnosis. The incidence of MANEC-P was relatively constant over the years, with an overall APC of −0.71. However, the age-adjusted incidence rate of MANEC-P APC was significantly different between 2001–2010 and 2010–2017 (7.89 vs. −11.13, *p* < 0.05) (Figure S3). Men had higher incidence rates than women during most of the study period. During 2010, males had a 1000% relative change from the 2001 MANEC-P incidence rate, moving from around 0.002 to more than 0.03 age-adjusted incidence rate. 

The incidence rates of MANEC-P were relatively constant throughout the months of the year in the range of 0.0007–0.001, with the highest cumulative incidence rate of cases reported in September and October. 

**Figure 3:**
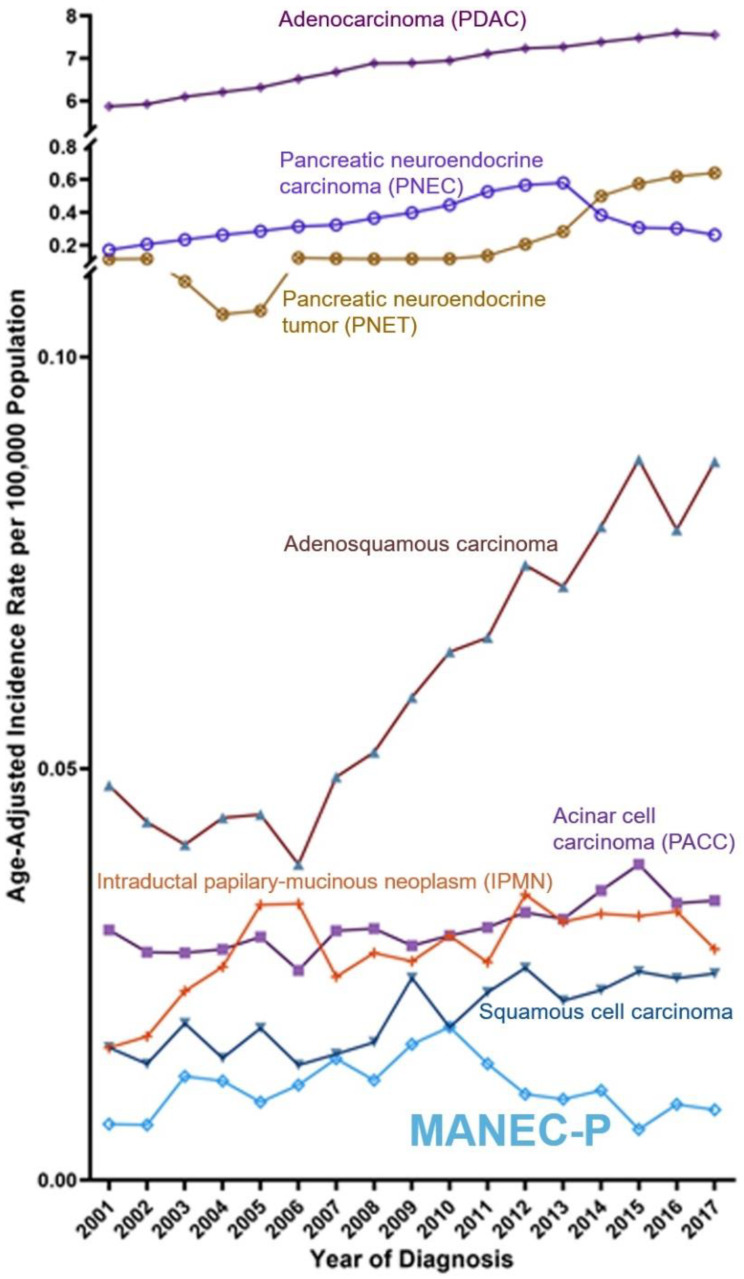
United States Temporal Trend over the time between 2001 and 2017 shows the age-adjusted incidence rate per 100,000 for each year of patients diagnosed with primary pancreatic cancer histological subtypes compared to MANEC-P [light blue line]. The *Y*-axis is not proportionate to show the pancreatic cancer histological subtypes with an age-adjusted incidence rate below 0.1.

Comparative Temporal Trend: The MANEC-P incidence rate was lower than that of all other pancreatic cancer subtypes, except for pancreatic carcinoma with neuroendocrine differentiation (Figure 3 and Figures S4 and S5). The peak incidence rates of MANEC-P in 2009 and 2010 were accompanied by a dramatic change in the incidence rate of pancreatic neuroendocrine tumors. 

**Figure 4:**
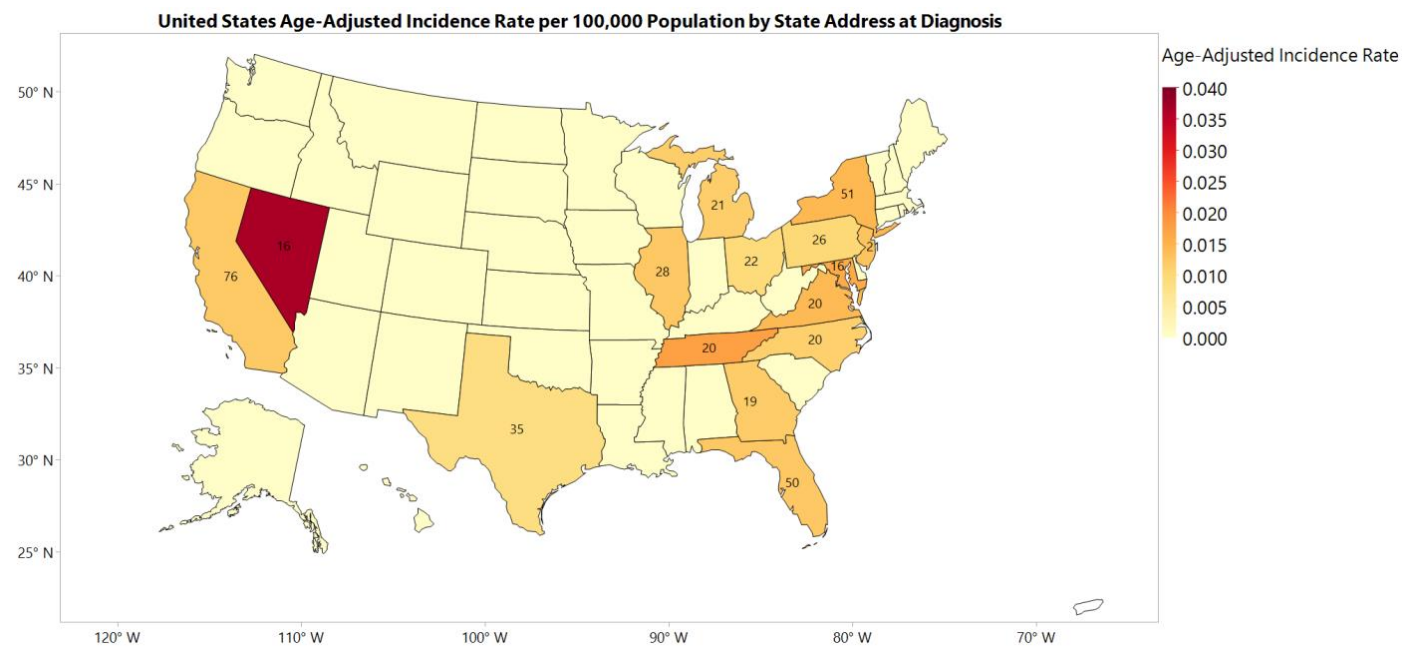
A map of the United States of America shows the state address at diagnosis of mixed pancreatic endocrine and exocrine tumor, malignancy, and patients colored by the age-adjusted incidence rate per 100,000 for each state between 2001 and 2017. The number inside each state represents the actual number of cases during the same duration. States colored with light yellow and without numbers in the map have a count number of less than 16 cases during the same period and the age-adjusted incidence rate for these states has not been calculated. One United States territory, Puerto Rico, has no colors because of a lack of information.

Geographic (Regional and State) Trend: Figure 4 and Figure S6 show the 2001–2017 incidence rates for each state and geographic region, respectively. 

Regionally, the northeastern region of the United States had the highest incidence rate of 0.013 per 100,000 population between 2001 and 2017. Compared to the northeast region of the US, the MANEC-P incidence rate was significantly lower in the midwest region (0.009, *p* = 0.008) and comparable without statistically significant differences in the southern and western regions (0.012 and 0.011, respectively, *p* > 0.05 for both). 

Among the states with reported MANEC-P incidence rates, Nevada had the highest incidence rate in the US. However, Nevada had a low cumulative number of cases compared to other states. The four most populous US states were California (*n* = 76), New York (*n* = 51), Florida (*n* = 50), and Texas (*n* = 35). 

### 3.2. Prevalence

The 17-year prevalence percentage of MANEC-P was 0.00005%, with 50 patients alive from the states reported in the SEER database. The projected 17-year limited-duration prevalence for MANEC-P in the US on 1 January 2018, was 189 patients. 

### 3.3. Cancer-Specific Survival (CSS) 

Descriptive CSS: The median CSS of patients with MANEC-P was estimated to be 29 (21, 51) months (Tables S2 and S3).

**Figure 5:**
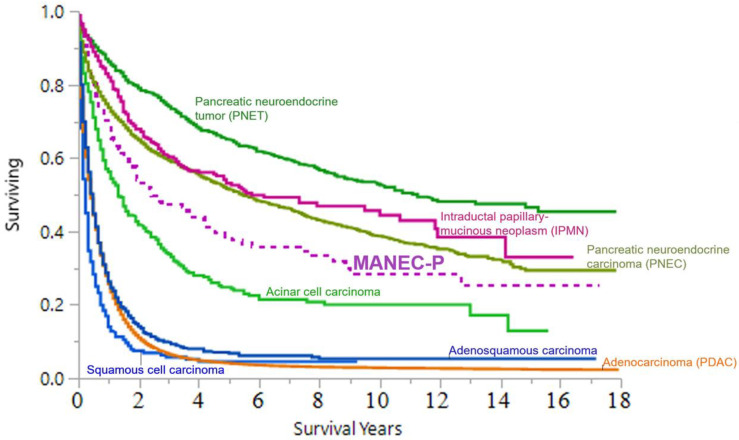
United States CSS over the time between 2001 and 2017 for MANEC-P patients compared with major pancreatic cancer histological subtypes.

Comparative CSS: Figure 5 and Figure S7 show the CSS of MANEC-P and the major pancreatic cancer and pancreatic neuroendocrine subtypes, including functional tumors, respectively. In comparison to the major pancreatic cancer subtypes, MANEC-P was the fifth-leading cause of pancreatic cancer-related death after adenocarcinoma, adenosquamous carcinoma, adenocarcinoma with mixed subtypes, and acinar cell carcinoma.

## 4. Discussion

MANEC-P is challenging to diagnose because of its mixed histologic morphology and phenotype, and the overall rarity of this pancreatic cancer subtype [2]. This study aimed to estimate the detailed epidemiology of MANEC-P in the US using large population-based databases, namely the NPCR and SEER databases. The age-adjusted incidence rate of MANEC-P in the US is approximately 10–12 new cases per 100 million population. During the last 20 years, the incidence rate has been relatively steady, peaking in 2010. Additionally, by the beginning of 2018, slightly fewer than 200 patients with MANEC-P were alive in the US. Although MANEC-P has the lowest incidence rate among all pancreatic cancer histopathological subtypes, it is associated with poor pancreatic cancer-related deaths compared with other pancreatic cancer subtypes. This study is the most comprehensive epidemiological analysis to date and highlights the need for new diagnostic and therapeutic strategies to improve the historically poor prognosis of neglected MANEC-P. 

The results of this study were not directly comparable to those of population-based studies previously published on pancreatic cancer subtypes, including the NCDB study by Pokrzywa et al. 2019 [11]. First, Pokrzywa et al. conducted their research using the National Cancer Database (NCDB), a facility-based registry that includes only new cases reported by the Commission on Cancer (CoC)-accredited cancer programs in the US and Puerto Rico, which covers approximately 70% of newly diagnosed cancers. Our study used the NPCR and SEER databases, which capture new incident cancers from all over the US. Second, Pokrzywa et al. excluded patients with metastatic disease (stage IV) while we included them. Finally, the NCDB dataset contains overall survival metrics. In our study, we were able to report cancer-specific survival in addition to other epidemiological measures, including incidence and prevalence. 

We were able to shed light on the demographic distribution of the patients with pancreatic MANEC-P. The incidence rates of MANEC-P were highest among the following categories: male, non-Spanish-Hispanic-Latino Hispanic, Black, and elderly patients. Male patients with MANEC-P had higher incidence rates by year of diagnosis and age. Moreover, most patients diagnosed with grade III MANEC-P were male. These differences in MANEC-P outcomes in relation to demographic differences are a rich area for healthcare disparity research among patients with MANEC-P and other pancreatic cancer subtypes. 

MANEC-P is frequently diagnosed in elderly patients and peaks in the 75–79 years-old patients’ group (0.061), particularly among men, while pancreatic cancer peaks among people aged 65–74 years, with a median age of 70 years at diagnosis [24,25]. The high incidence rate among elderly patients may be due to the difficulty in establishing a MANEC-P diagnosis, thus delaying its accurate identification, and shifting the incidence peak towards the elderly. The decrease in the incidence rate of MANEC-P after its peak followed the general trend in age-adjusted cancer incidence rates due to age-related death. 

Patients with MANEC-P often present at a late stage with evidence of distant metastasis, which differs from the presentation of PDAC for multiple reasons. First, the lack of pancreatic cancer screening programs and specific diagnostic biomarkers for MANEC-P contribute to its delayed diagnosis. Additionally, late-stage MANEC-P presentation can be related to the late development of symptoms, as the neuroendocrine component of MANEC-P is non-functional, thus delaying patient symptoms compared with patients with functional neuroendocrine tumors. In addition, the challenges in diagnosing MANEC-P (i.e., adequacy of the biopsy tissue sample for staining, need for multiple staining, quality assurance for pathology when using population databases, and insufficient literature on steps for diagnosis) may cause misdiagnosis or result in no definitive diagnosis. In turn, these patients present with systemic disease at the time of an accurate diagnosis. Indeed, an accurate diagnosis may only be possible during the autopsy, as shown in some SEER cases. The late-stage presentation may also change the treatment strategy to palliative therapy, thereby affecting the outcomes of patients with MANEC-P. Late presentation contributes to the poor prognosis of MANEC-P with the distant disease compared to localized MANEC-P (Table S3). Notably, metastatic PDAC had a poorer prognosis compared to our data on metastatic MANEC-P (5-year CSS = 18.7%, and 10-year CSS = 12.5%), similar to the overall CSS trend (Figure 5).

MANEC-P is largely reported as the sole primary tumor; however, in some instances, it is reported as one of two or one of three reported tumors. One possible explanation for this variability is that MANEC-P is a component of hereditary cancer syndromes. Alternatively, MANEC-P formation could result from oncogene or tumor suppressor gene mutations in other concurrent cancers. 

The incidence of MANEC-P has remained relatively constant over the last two decades, with a surge in 2010. In 2010, men had a 1000% relative increase from the 2001 MANEC-P incidence rate. No apparent reason for this significant increase is identified. However, these data were obtained from the cancer registry database and may have been affected by changes in coding over time. Although the code for MANEC-P has not changed over the years, the WHO introduced a new classification system for PNET in 2010 [26,27]. It is possible that this new classification system dramatically increased the incidence rate of all PNETs in the US and concomitantly, the incidence rate of MANEC-P. 

Many factors could impact the geographical distribution of MANEC-P, including the demographic differences in each state [28,29]. More than one-quarter of the seniors aged > 65 years lived in the four states with the highest cumulative MANEC-P cases: California, Florida, Texas, and New York [30,31]. Moreover, New York has one of the highest overall incidence rates of pancreatic cancer. Nevada has the highest incidence rate of MANEC-P in the US. Notably, Nevada had a higher cancer incidence rate than the remaining US. The reason for the high incidence rate in Nevada is unknown. However, this high incidence rate was not driven by the high number of cumulative cases, as Nevada had relatively medium cumulative MANEC-P incidents with a relatively small population compared to the other states.

The geographical distribution of MANEC-P may help alleviate its tumor burden in two strategic directions. First, by identifying state-by-state MANEC-P incidence rates, these data can be used to efficiently allocate resources toward state-level cancer diagnostics for early detection and accurate diagnosis of MANEC-P. Second, a large collaborative natural history study facilitated through the identification of states most affected by MANEC-P and reaching out to living patients (i.e., around 189 MANEC-P patients projected alive by 2018), for further applicable translational and clinical research on MANEC-P. These strategies will provide a foundation for future multimodal strategic interventions such as advanced surgery and the development of targeted therapies.

Comparative epidemiology of MANEC-P is essential. We were able to show that MANEC-P represents one of the leading causes of pancreatic cancer mortality in the US and is responsible for more deaths than PNEC and PNET, something that has not been previously demonstrated. This finding bridges an important gap in current knowledge, as MANEC-P-specific fatalities were previously unknown. Current scientific methodology (i.e., basic, clinical, and translational) is poised to answer the many difficult questions that lie ahead about MANEC-P’s origin and natural history, help elucidate potential therapeutics, and discern adequate responses to therapy.

The SEER coding is primarily dependent on the ICD-O coding system. This system facilitates documentation of data entry in state-run cancer registries to allow meaningful data comparisons. However, histological coding for rare cancer types may not be fully developed without quality control of rare tumor pathological assessments. Although the ICD-O is frequently updated [2], the ICD-O coding for rare cancer subtypes is unclear. Two illustrative examples of perplexing codes are presented in Table S1. The first example included codes for adenocarcinoma and endocrine carcinomas, resulting in three separate ICD-O-3 codes: 8154/3, 8244/3, and 8574/3. In the second example, some ICD-O-3 codes included multiple cancer subtypes: 8154/3 and 8255/3. These examples illustrate the limitations and potential inconsistencies of these cancer registries for rare cancer subtypes, which may underestimate the epidemiological measures of rare diseases owing to a lack of familiarity with such a rare malignancy.

This study has limitations. The SEER database does not encompass the entire US, and the dataset used in the analysis was limited to 18 states to estimate MANEC-P prevalence and CSS. However, we included the NPCR database, which includes the entire US, to estimate the incidence and prevalence outcomes. One of the limitations of using population databases for rare cancers is the lack of quality assurance regarding pathological assessment. We tried to minimize this by including only patients with positive histology and excluding those without histopathological confirmation. The ICD-O-3 histology and behavior codes for mixed pancreatic neuroendocrine (islet) and non-neuroendocrine carcinoma did not distinguish between non-neuroendocrine components (i.e., adenocarcinoma, acinar, or both). Fortunately, the diagnosis code for this disease has remained the same since the inception of SEER in 1975. However, the name of the condition was changed a few times. Therefore, we focused our search on using the same ICD-O-3 topographic code (8154/3) and pancreatic anatomical site for diagnosis to ensure diagnostic accuracy.

## 5. Conclusions

MANEC-P of the pancreas is a rare cancer associated with poor pancreatic cancer-related deaths in the US compared with other pancreatic cancer subtypes. Currently, no standard of care is available for this fatal cancer. This study helps establish epidemiological benchmarks for MANEC-P in the US, including data on incidence, prevalence, and CSS. These data will be used to create a MANEC-P-specific working group/consortium to combine data to generate real-world evidence. A MANEC-P patient advocacy group should be created to consider their opinions in future MANEC-P-related research. A large natural history study of MANEC-P is needed to further understand this cancer and discover how current medical and surgical therapies can optimize the oncologic outcomes of MANEC-P.

## Figures and Tables

**Table 1:** MANEC-P Incidence Rate by demographics, tumor characteristics and temporal trend.

Variable	Level	MANEC-P Cases, *n* (%)	Age-Adjusted Incidence Rate per 10,000,000 (95% CI)	*p*-Value
Program	NPCR	611 (96.98)	1.13 (1.04–1.23)	Reference
	SEER	19 (3.02)	0.98 (0.58–1.54)	0.64
Age, years	00–39 years	37 (5.87)	0.14 (0.1–0.19)	Reference
	40–44 years	28 (4.44)	0.77 (0.51–1.11)	<0.05
	45–49 years	35 (5.56)	0.95 (0.66–1.32)	<0.05
	50–54 years	62 (9.84)	1.73 (1.32–1.73)	<0.05
	55–59 years	69 (10.95)	2.14 (1.66–2.14)	<0.05
	60–64 years	82 (13.02)	3.06 (2.44–3.06)	<0.05
	65–69 years	81 (12.86)	3.80 (3.02–3.8)	<0.05
	70–74 years	85 (13.49)	5.14 (4.11–5.14)	<0.05
	75–79 years	97 (15.40)	6.09 (4.82–6.09)	<0.05
	80–84 years	51 (8.10)	5.31 (3.95–5.31)	<0.05
	85+ years	21 (3.33)	2.30 (1.43–2.30)	<0.05
Sex	Male	357 (56.67)	1.30 (1.25–1.55)	Reference
	Female	273 (43.33)	0.92 (0.81–1.04)	<0.05
Age, years; and Sex				
00–39 years	Female	18 (48.65)	0.13 (0.08–0.21)	Reference
	Male	19 (51.35)	0.14 (0.08–0.22)	1
40–44 years	Female	^	^	^
	Male	^	^	^
45–49 years	Female	^	^	Reference
	Male	20	1.09 (0.67–1.69)	^
50–54 years	Female	24 (38.71)	1.31 (0.84–1.95)	Reference
	Male	38 (61.29)	2.16 (1.53–2.96)	0.07
55–59 years	Female	31 (44.93)	1.87 (1.27–2.65)	Reference
	Male	38 (55.07)	2.43 (1.72–3.33)	0.33
60–64 years	Female	34 (41.46)	2.43 (1.69–3.40)	Reference
	Male	48 (58.54)	3.75 (2.76–4.97)	0.07
65–69 years	Female	33 (40.74)	2.92 (2.01–4.10)	Reference
	Male	48 (59.26)	4.79 (3.53–6.35)	0.04
70–74 years	Female	39 (45.88)	4.33 (3.08–5.92)	Reference
	Male	46 (54.12)	6.11 (4.47–8.14)	0.14
75–79 years	Female	34 (43.04)	4.62 (3.20–6.45)	Reference
	Male	45 (56.96)	8.03 (5.85–0.74)	0.02
80–84 years	Female	25 (49.02)	4.32 (2.80–6.38)	Reference
	Male	26 (50.98)	6.80 (4.44–9.96)	0.14
85+ years	Female	^	^	Reference
	Male	16	5.39 (3.08–8.76)	^
Ethnicity	Spanish-Hispanic-Latino	41 (6.51)	0.80 (0.56–1.09)	Reference
	Non-Spanish-Hispanic-Latino	589 (93.49)	1.16 (1.07–1.26)	0.021
Race	White	525 (83.33)	1.12 (1.02–1.22)	Reference
	Black	78 (12.38)	1.30 (1.02–1.64)	0.24
	Asian or Pacific Islander	20 (3.17)	0.89 (0.54–1.39)	0.38
	American Indian/Alaska Native	^	^	^
	Other/Unknown	^	^	^
Primary site, pancreas	Head	245 (38.83)	0.44 (0.38–0.50)	Reference
	Body, tail, and duct	249 (39.62)	0.45 (0.40–0.51)	0.87
	IOL	16 (2.54)	0.03 (0.02–0.04)	<0.01
	Other specified parts and overlapping lesions	44 (6.97)	0.07 (0.06–0.10)	<0.01
	NOS	76 (12.04)	0.14 (0.11–0.17)	<0.01
Grade	Grade I	117 (18.57)	0.21 (0.17–0.25)	Reference
	Grade II	126 (20.00)	0.22 (0.18–0.26)	0.80
	Grade III	123 (19.52)	0.22 (0.18–0.26)	0.76
	Grade IV	19 (3.02)	0.03 (0.02–0.05)	<0.01
	Unknown	245 (38.89)	0.44 (0.39–0.50)	<0.01
Historical Staging	In situ/Localized disease	123 (19.52)	0.23 (0.19–0.27)	Reference
	Regional, direct extension only	96 (15.24)	0.17 (0.14–0.21)	0.06
	Regional, regional lymph nodes only	52 (8.25)	0.09 (0.14–0.21)	<0.01
	Regional direct extension and regional lymph nodes	122 (19.37)	0.22 (0.18–0.26)	0.78
	Regional, NOS	^	^	^
	Distant site(s)/node(s) involved	219 (34.76)	0.39 (0.34–0.45)	<0.01
	Not applicable	^	^	^
	Unknown/unstaged/unspecific	18 (2.86)	0.03 (0.02–0.05)	<0.01
Multiple primary sites	One primary only	475 (75.40)	0.85 (0.78–0.93)	Reference
	1st of 2 or more primaries	46 (7.30)	0.08 (0.06–0.11)	<0.01
	2nd of 2 of more primaries	89 (14.13)	0.16 (0.13–0.19)	<0.01
	3rd of 3 or more primaries	16 (2.54)	0.03 (0.02–0.05)	<0.01
Regions	Northeast	132 (20.95)	1.25 (1.04–1.49)	Reference
	Midwest	110 (17.46)	0.88 (0.72–1.06)	<0.01
	South	250 (39.68)	1.21 (1.06–1.37)	0.82
	West	138 (21.91)	1.13 (0.95–1.34)	0.45

^ Statistics not displayed due to fewer than 16 cases. Rates are per 10,000,000 and age-adjusted to the 2000 US standard population. The total patients were 611 from NPCR patients and an additional 19 from SEER.

## Data Availability

Research data supporting this publication are available through the Surveillance, Epidemiology, and End Results (SEER) Program (www.seer.cancer.gov) SEER*Stat Database. This study included all patients diagnosed with MANEC-P in the United States. For incidence, patients were included between 2001 and 2017 through the following dataset: “NPCR and SEER Incidence–U.S. Cancer Statistics 2001–2017 Public Use Research Database, 2019 Submission (2001–2017), US Department of Health and Human Services, Centers for Disease Control and Prevention (CDC) and National Cancer Institute (NCI), released in June 2020 and accessed at www.cdc.gov/cancer/uscs/public-use, accessed on 12 August 2020.

## Supplementary Materials

The following supporting information can be downloaded at: https://www.mdpi.com/article/10.3390/cancers15030840/s1, Table S1. pancreatic cancer subtypes specific ICD-O-3 histology and behavior codes. Table S2. United States CSS in months between 2001 and 2017 for MANEC-P patients compared to other pancreatic cancer histological subtypes. Table S3. MANEC-P CSS by Historical Staging. Figure S1. MANEC-P incidence rate distribution by sex and age groups. Figure S2. MANEC-P incidence rate by grade and sex. Figure S3. United States Annual Percentage Change (APC) of the age-adjusted incidence rate per 100,000 before and after 2010 for each year of patients diagnosed with mixed pancreatic endocrine and exocrine tumor, malignant. The (*) indicates that the APC is significantly different from zero at the alpha = 0.05 level. The overall APC is −0.71. Figure S4. United States Temporal Trend over the time between 2001 and 2017 shows the age-adjusted incidence rate per 100,000 for each year of patients diagnosed with pancreatic cancer histological subtypes compared to the mixed pancreatic endocrine and exocrine tumor, malignant (MANEC-P) [light blue line]. The *Y*-axis is not proportionate to show the pancreatic cancer histological subtypes with an age-adjusted incidence rate below 0.1. Figure S5. United States temporal trends between 2001 and 2017 show the age-adjusted incidence rate per 100,000 for each year of patients diagnosed with pancreatic cancer with neuroendocrine components including the functional PNET histological subtypes in comparison to MANEC-P [light blue line]. The *Y*-axis is not proportionate to show the pancreatic cancer histological subtypes, with an age-adjusted incidence rate below 0.1. Figure S6. MANEC-P age-adjusted incidence rate by the United States of America regional map between 2001 and 2017. Figure S7. United States CSS over the time between 2001 and 2017 for MANEC-P patients compared with pancreatic neuroendocrine tumor subtypes.
