# Supplementary material for: Mixed Acinar Neuroendocrine Carcinoma of the Pancreas: Comparative Population-Based Epidemiology of a Rare and Fatal Malignancy in The United States
Source: Cancers (Basel). 2023 Jan 30;15(3):840. doi: 10.3390/cancers15030840 (PMC9913846; doi:10.3390/cancers15030840)
Supplement: Supplementary file 1 [file cancers-15-00840-s001.zip › cancers-2164759-supplementary.pdf]

Supplementary Materials: The following supporting information can be downloaded at: www.mdpi.com/xxx/s1, **Table S1.** pancreatic cancer subtypes specific ICD-O-3 histology and behavior codes. **Table S2.** United States CSS in months between 2001 and 2017 for MANEC-P patients compared to other pancreatic cancer histological subtypes. **Table S3.** MANEC-P CSS by Historical Staging. **Figure S1.** MANEC-P incidence rate distribution by sex and age groups. **Figure S2.** MANEC-P incidence rate by grade and sex. **Figure S3.** United States Annual Percentage Change (APC) of the age-adjusted incidence rate per 100,000 before and after 2010 for each year of patients diagnosed with mixed pancreatic endocrine and exocrine tumor, malignant. The (*) indicates that the APC is significantly different from zero at the alpha = 0.05 level. The overall APC is – 0.71. **Figure S4.** United States Temporal Trend over the time between 2001 and 2017 shows the age-adjusted incidence rate per 100,000 for each year of patients diagnosed with pancreatic cancer histological subtypes compared to the mixed pancreatic endocrine and exocrine tumor, malignant (MANEC-P)[light blue line]. The Y-axis is not proportionate to show the pancreatic cancer histological subtypes with an age-adjusted incidence rate below 0.1. **Figure S5.** United States temporal trends between 2001 and 2017 shows the age-adjusted incidence rate per 100,000 for each year of patients diagnosed with pancreatic cancer with neuroendocrine components including the functional PNET histological subtypes in comparison to MANEC-P [light blue line]. The Y-axis is not proportionate to show the pancreatic cancer histological subtypes, with an age-adjusted incidence rate below 0.1. **Figure S6.** MANEC-P age-adjusted incidence rate by the United States of America regional map between 2001 and 2017. **Figure S7.** United States CSS over the time between 2001 and 2017 for MANEC-P patients compared with pancreatic neuroendocrine tumors subtypes.

Table S1. Pancreatic cancer subtypes specific ICD-O-3 histology and behavior codes.

ICD-O-3 Code	Diagnosis	Solid Tumor Main Site
8453/3	Intraductal papillary-mucinous neoplasm (IPMN)	
8470/3	Mucinous cystadenocarcinoma, NOS	
8471/3	Papillary mucinous cystadenocarcinoma	
8140/3	Adenocarcinoma, NOS (PDAC)	
8147/3	Basal cell adenocarcinoma	
8070/3	Squamous cell carcinoma, NOS	
8560/3	Adenosquamous carcinoma	
8244/3	Mixed Adenocarcinoma with neuroendocrine carcinoma (Composite carcinoid)	Colon and then others
8574/3	Adenocarcinoma with neuroendocrine differentiation	Colon and then others
8255/3	Adenocarcinoma with mixed subtypes (3 tumors: adenocarcinoma with two or more of the following: papillary, clear cell, mucinous, or signet)	Breast, Colon, Lung, and then others
8150/3	Pancreatic endocrine tumor, malignant (PNET)	
8151/3	Insulinoma, malignant	
8152/3	Glucagonoma, malignant	
8153/3	Gastrinoma, malignant	
8154/3	Mixed pancreatic endocrine and exocrine tumor, malignant	
8155/3	Vipoma, malignant	
8156/3	Somatostatinoma, malignant	
8240/3	Carcinoid tumor, NOS	We combined it with 8150/3 (PNET) as it probably has been

		used interchangeably
8013/3	Large cell neuroendocrine carcinoma	Esophagus and then others
8041/3	Small cell carcinoma, NOS	Esophagus and then others
8246/3	Neuroendocrine carcinoma, NOS (PNEC)	
8550/3	Acinar cell carcinoma	
8551/3	Acinar cell cystadenocarcinoma	

Table S2. United States CSS in months between 2001 and 2017 for MANEC-P patients compared to other pancreatic cancer histological subtypes.

Group	Median Time	Lower 95%	Upper 95%
**8154/3: Mixed pancreatic endocrine and exocrine tumor, malignant**	**29**	**21**	**51**
8151/3: Insulinoma, malignant	.	.	.
8156/3: Somatostatinoma, malignant	.	.	.
8153/3: Gastrinoma, malignant	150	82	.
8150/3: Pancreatic endocrine tumor, malignant	135	111	.
8152/3: Glucagonoma, malignant	126	64	.
8453/3: Intraductal papillary-mucinous neoplasm (IPMN)	69	54	128
8246/3: Neuroendocrine carcinoma, NOS	66	60	71
8155/3: Vipoma, malignant	46	16	193
8244/3: Mixed adenoneuroendocrine carcinoma (ICD-O-3 update)	32	6	32
8470/3: Mucinous cystadenocarcinoma, NOS	28	20	39
8550/3: Acinar cell carcinoma	16	13	20
8255/3: Adenocarcinoma with mixed subtypes	14	10	19
8574/3: Adenocarcinoma with neuroendocrine differentiation	10	5	15
8013/3: Large cell neuroendocrine carcinoma	9	8	13
8560/3: Adenosquamous carcinoma	6	5	6
8041/3: Small cell carcinoma, NOS	5	3	6
8140/3: Adenocarcinoma, NOS	5	.	.
8070/3: Squamous cell carcinoma, NOS	3	2	4
Combined	6	.	.

Table S3. MANEC-P Cancer-Specific Survival by Historical Staging.

MANEC-P Tumor Characteristics (n)	Median survival, months	Survival Rate, %			
		1-year	3-year	5-year	10-year
All (145)	29.22	70	45.5	37.9	26.8
Localized only (17)	15	94.1	87.8	87.8	58.2
Regional (56)	33.89	78.1	51.7	41.9	36.3
Distant (49)	12.24	52.3	22.4	18.7	12.5
Unknown (23)	32.13	68.3	45.5	30.3	20.2

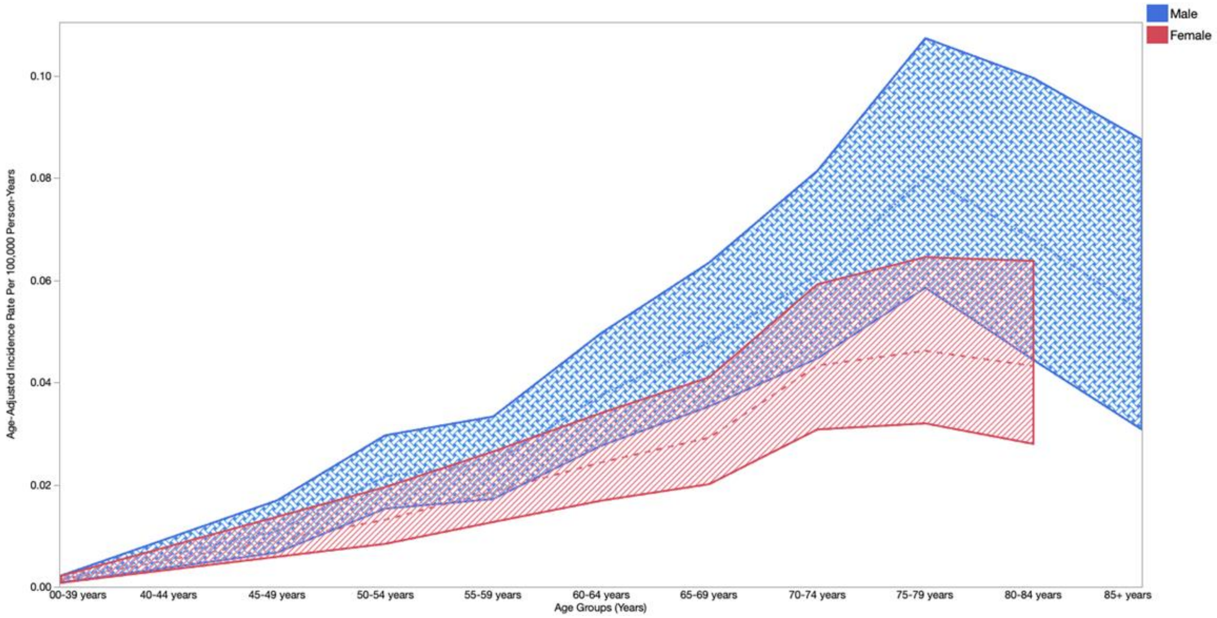

Figure S1. MANEC-P incidence rate distribution by sex and age groups.

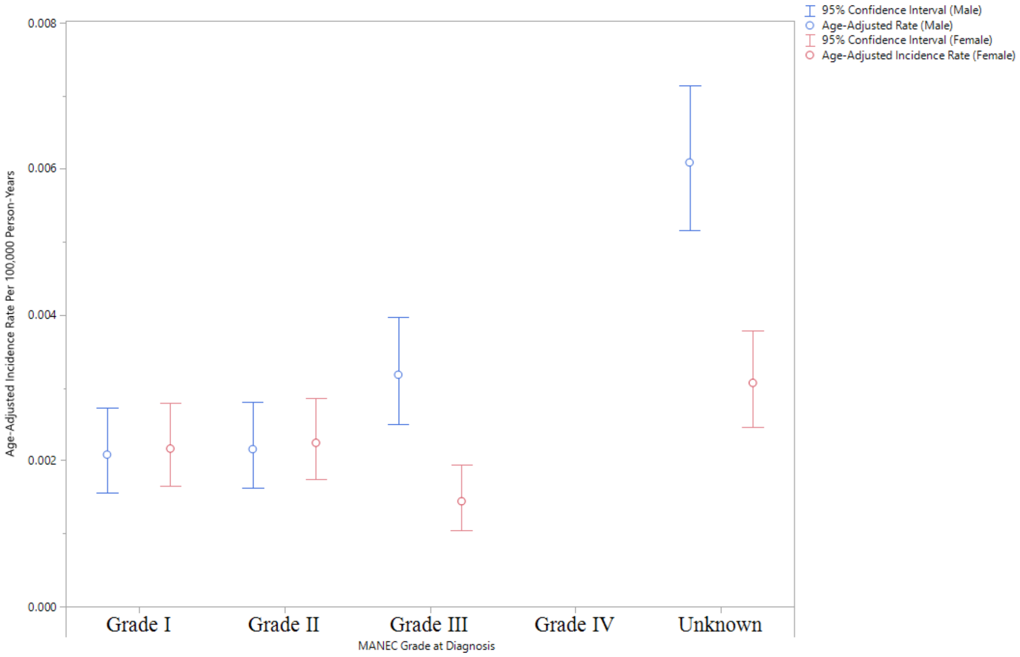

Figure S2. MANEC-P incidence rate by grade and sex.

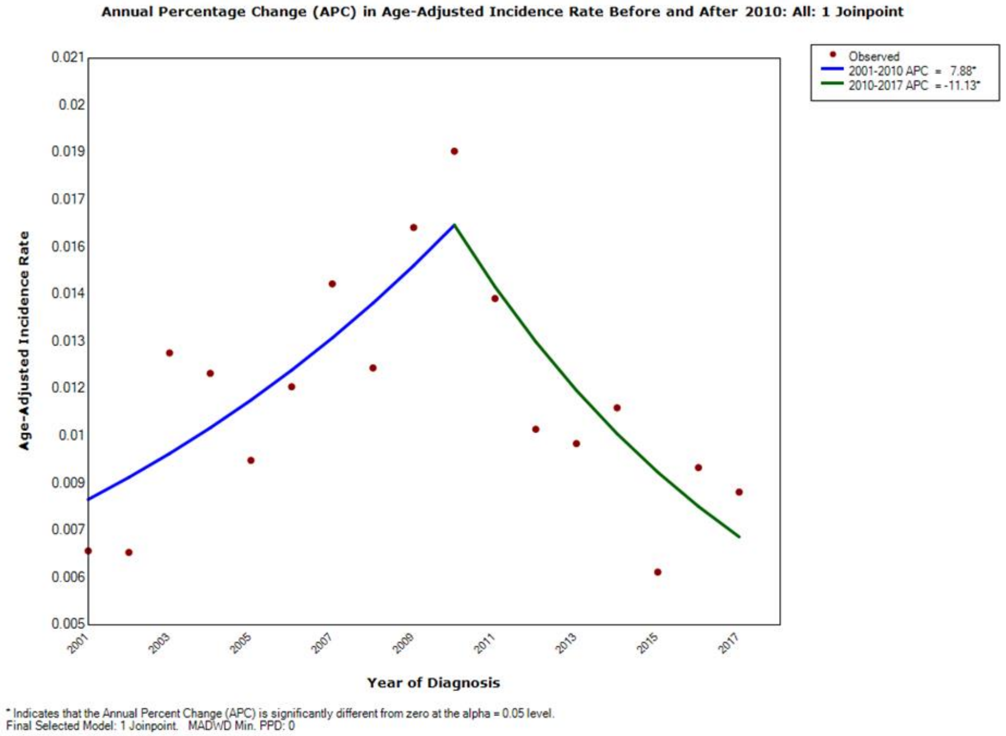

Figure S3. United States Annual Percentage Change (APC) of the age-adjusted incidence rate per 100,000 before and after 2010 for each year of patients diagnosed with mixed pancreatic endocrine and exocrine tumor, malignant. The (*) indicates that the APC is significantly different from zero at the alpha = 0.05 level. The overall APC is – 0.71.

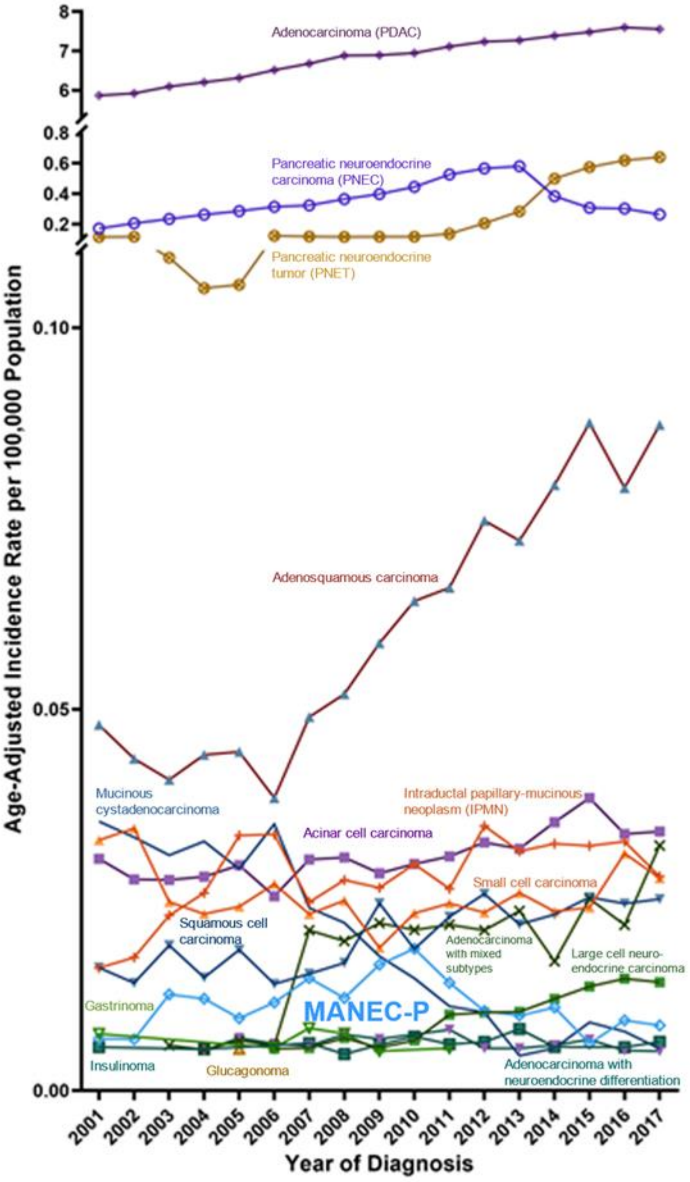

Figure S4. United States Temporal Trend over the time between 2001 and 2017 shows the age-adjusted incidence rate per 100,000 for each year of patients diagnosed with pancreatic cancer histological subtypes compared to the mixed pancreatic endocrine and exocrine tumor, malignant (MANEC-P)[light blue line]. The Y-axis is not proportionate to show the pancreatic cancer histological subtypes with an age-adjusted incidence rate below 0.1.

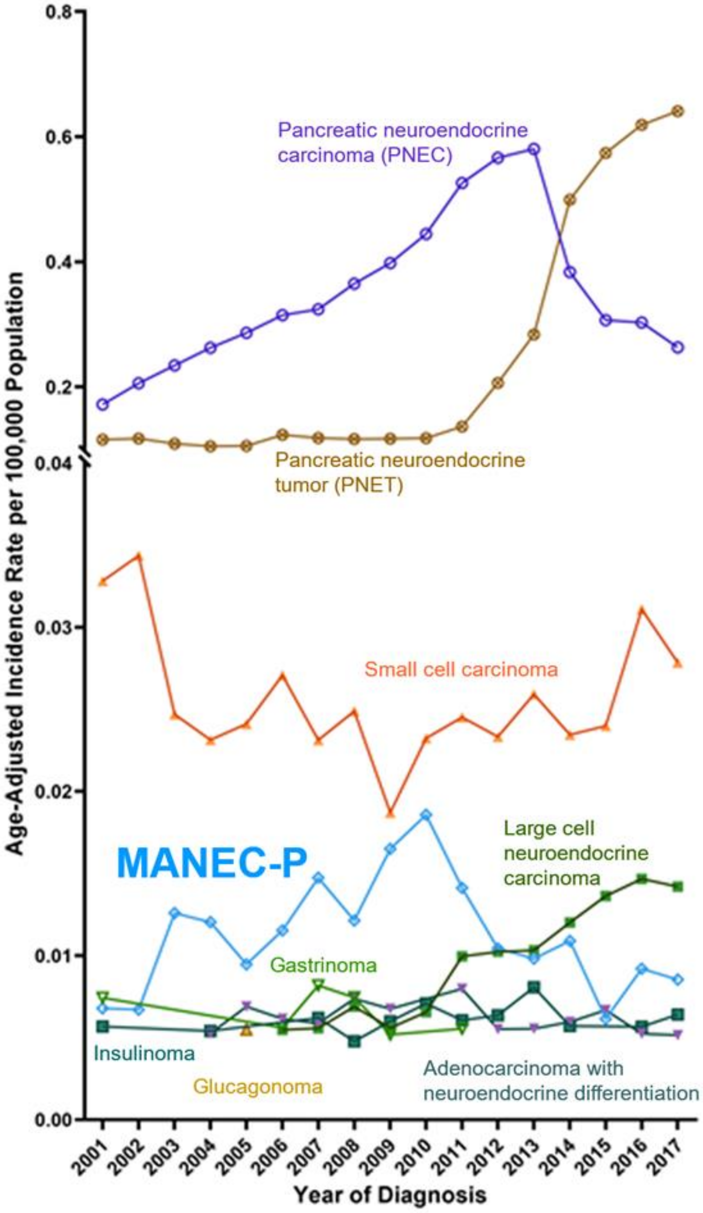

Figure S5. United States temporal trends between 2001 and 2017 shows the age-adjusted incidence rate per 100,000 for each year of patients diagnosed with pancreatic cancer with neuroendocrine components including the functional PNET histological subtypes in comparison to MANEC-P [light blue line]. The Y-axis is not proportionate to show the pancreatic cancer histological subtypes, with an age-adjusted incidence rate below 0.1.

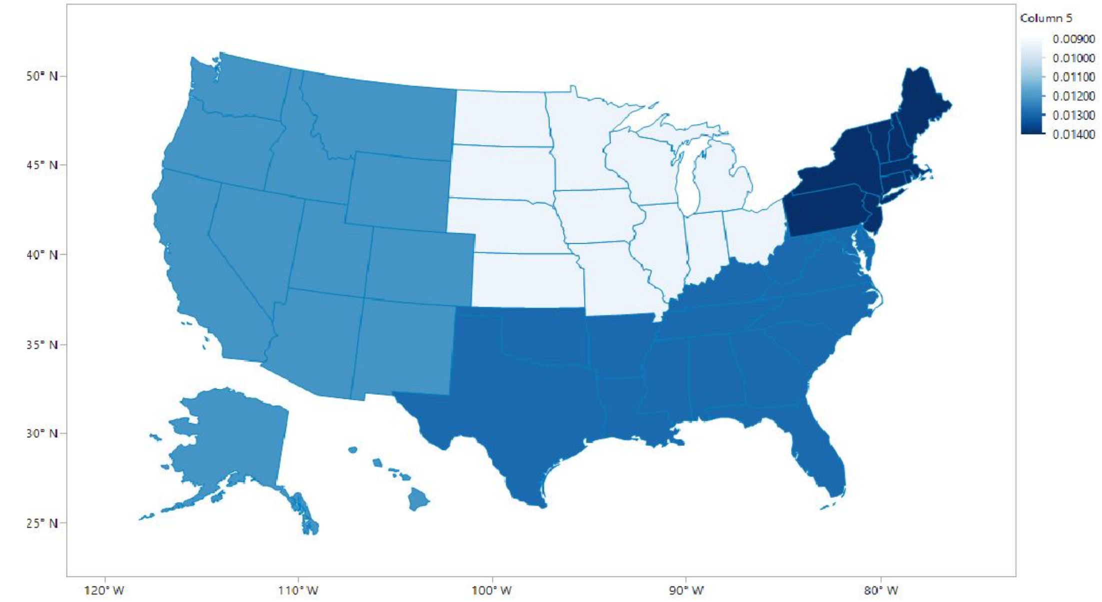

Figure S6. MANEC-P age-adjusted incidence rate by the United States of America regional map between 2001 and 2017.

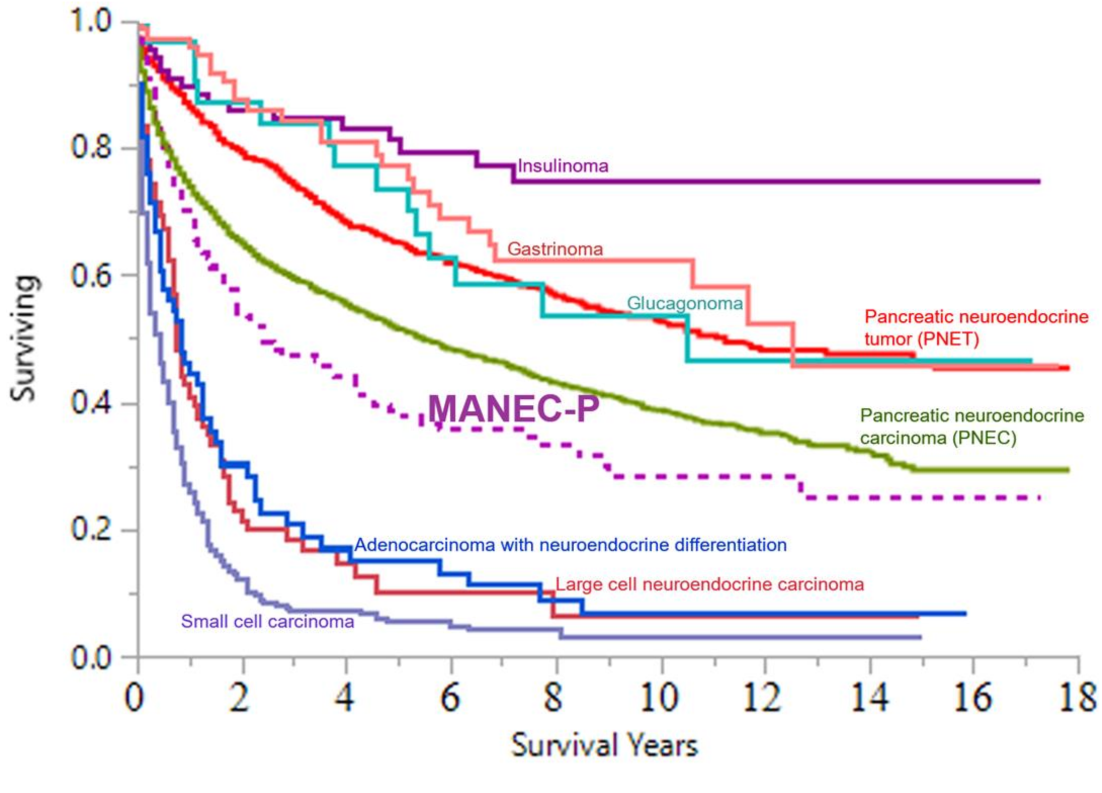

Figure S7. United States CSS over the time between 2001 and 2017 for MANEC-P patients compared with pancreatic neuroendocrine tumors subtypes.
